# Supplementary material for: Evidence-based practice and its associated factors among point-of-care nurses working at the teaching and specialized hospitals of Northwest Ethiopia: A concurrent study
Source: PLoS One. 2022 May 5;17(5):e0267347. doi: 10.1371/journal.pone.0267347 (PMC9070954; doi:10.1371/journal.pone.0267347)
Supplement: S1 Appendix — (DOCX) [file pone.0267347.s001.docx]

**S1 Appendix. Research Questionnaire:**

**Instruction letter**

Dear participants, Thank you for your willingness to participate in this study which aims to assess Evidence-Based Practice and its associated factors among Nurses. This questionnaire has nine sections; Scio-demographic data, Nurses Evidence-Based Practice, Knowledge on Evidence-Based Practice, Nurses Preferred Sources of Health Information, Awareness about Electronic Information Sources, information searching skills, attitude towards Evidence-Based Practice, self-efficacy for Evidence-Based Practice, and factors affecting Evidence-Based Practice. You are required to have read each question carefully and give the answer you think correct for yourself by making a circle, fill or tick (√) accordingly.

If you have any inquiry regarding the study, please contact on the following address.

PI Phone No: +251916248311/+251918813287

Email: abebirhanu21@gmail.com

Thank you.

**Section I: Scio-demographic Data**

| **S.N** | **Questions** | **Choices** | **skip** |
| --- | --- | --- | --- |
| 101 | Age in years | ________years |  |
| 102 | Gender | 1. Male 2. Female |  |
| 103 | Educational status | 1. BSc Degree  2. MSc Degree  3. Ph.D. Degree |  |
| 104 | Work experience in years | _______ years |  |
| 105 | Current place of work | 1. The University of Gondar teaching Specialized Referral hospital 2. Tibebe Gion teaching Specialized Referral hospital |  |
| 106 | Current working unit | 1. Inpatient departments 2. ICU wards 3. OR 4. Emergency wards 5. Outpatient Departments |  |
| 107 | Worker position | 1. Staff nurse  2. Team leader Nurse  3. Admin nurse |  |
| 108 | Have you ever received any training related to Evidence-Based Practice? | 1. Yes  2. No |  |
| 109 | When do you get the training? | 1. Undergraduate 2. Postgraduate | If your answer for Q108 is “No” go to Q110 |
| 110 | Which type of electronic device do you use in searching for evidence? | 1) Low-end phones or basic phones (*Have only core functionalities (voice calling and SMS messaging))*  2) Feature phones or internet-enabled phones (*can access the internet for sending an email, browsing the web and so on (but usually without the same ease-of-use as smartphones due to smaller screens, etc.)*  3) Smartphone (*provide voice, SMS, and internet access which can run built-in applications for a wide variety of purposes (e.g., Web browsing, calendars, document reading, and others).*  4) Tablet computer  5) Laptop Computer  6) Desktop computer |  |
| 111 | Do you have internet access in your home? | 1) Yes  2) No | If your answer of Q110 is not 5 or 6 jump Q111 and go to the next |

**Section II: Nurses Evidence-Based Practice**

| **S.N** | **Question** | **Choices** | | | |
| --- | --- | --- | --- | --- | --- |
|  |  | **Never** | **Monthly** | **Weekly** | **Daily** |
| 201 | I formulate an answerable PICO question (P: patient, interest group, nursing problem, I: new intervention or nursing method for patient, C: existing nursing intervention or comparison to compare, O: expected outcome of interest)  (**Ask**) | 1 | 2 | 3 | 4 |
| 202 | I conduct online searches or tracked down the relevant evidence once I have formulated the PICO questions using databases and/or search engines (CINAHL, Medline, PubMed, etc.) (**Acquire**) | 1 | 2 | 3 | 4 |
| 203 | I can rapidly appraise studies to determine their applicability (usefulness in own clinical practice) and the validity (closeness to the truth) of the research evidence to my clinical practice (**Appraise**) | 1 | 2 | 3 | 4 |
| 204 | I Relate research findings to my clinical practice and point out similarities and differences (**Aggregate**) | 1 | 2 | 3 | 4 |
| 205 | I integrate the most up-to-date results from scientific research with my clinical experiences to solve problems related to my professional practice (**Apply**) | 1 | 2 | 3 | 4 |
| 206 | I collect patient outcome data to evaluate how well I achieved my nursing care plan and my evidence-based practices (**Assess**) | 1 | 2 | 3 | 4 |
| 207 | Once I have implemented a change and evaluate its effectiveness I share my findings and or outcomes with others in my facility. (**Announce**/**appearance)** | 1 | 2 | 3 | 4 |

**Section III: Nurses knowledge about Evidence-Based Practice**

| **S.N** | **Questions** | Yes | No | I don’t know |
| --- | --- | --- | --- | --- |
| 301 | Evidence-Based Practice is the integration of best research evidence, clinical expertise, and patient's values and preferences in making decisions about the care of individual patients. | 1 | 0 | 9 |
| 302 | Using Evidence-Based Practice increases the certainty that the selected treatment or nursing procedure will be effective | 1 | 0 | 9 |
| 303 | Do you know Evidence-based practice is a process of making decisions using information derived from current scientifically proven evidence? | 1 | 0 | 9 |
| 304 | Do you know Evidence-based practice involves a series of steps from identifying the clinical question, finding the answer/evidence, assessing the validity of evidence, to applying it if clinically suitable? | 1 | 0 | 9 |
| 305 | Evidence-based practice requires the use of critical appraisal skills to ensure the quality of all the research papers retrieved | 1 | 0 | 9 |
| 306 | A literature search using Boolean operator (“OR”, “AND”, “NOT” or “NEAR”) would reduce the number of citations that the search would produce | 1 | 0 | 9 |
| 307 | The best and quickest way to find evidence is by reading textbooks. | 1 | 0 | 9 |
| 308 | Previous clinical experience is more important than research findings in choosing the best treatment available for a patient | 1 | 0 | 9 |
| 309 | Understanding of patient’s preferences is essential for identifying the best available treatment for that particular patient | 1 | 0 | 9 |
| 310 | Do you understand and demonstrates the commonly used statistical terms (odds ratio, confidence interval, chance, bias, confounding variables, etc.) covered in the paper? | 1 | 0 | 9 |

**Section IV: Nurses Preferred Sources of Health Information**

|  | Questions | Never | Rarely  (once in  a few  months) | Sometimes  (at least  once a  month) | Often  (once a  week) | Always  (several  times a  Day) |
| --- | --- | --- | --- | --- | --- | --- |
| 401 | Reading printed research articles, magazines, pamphlets | 1 | 2 | 3 | 4 | 5 |
| 402 | Referring to clinical practice from medical journals (HINARI...) | 1 | 2 | 3 | 4 | 5 |
| 403 | Reading articles from searching of electronic databases with Internet (e.g. CINAHL, Medline) | 1 | 2 | 3 | 4 | 5 |
| 404 | Referring medical apps (e.g. Up-to-Date, Medscape) | 1 | 2 | 3 | 4 | 5 |
| 405 | Referring policy and guideline manuals | 1 | 2 | 3 | 4 | 5 |
| 406 | Information learning from training | 1 | 2 | 3 | 4 | 5 |
| 407 | Information sharing from colleagues | 1 | 2 | 3 | 4 | 5 |

**Section V: Awareness about Electronic Information Sources used for getting Best evidence for Evidence-Based Practice**

| No | Questions | Unaware | Aware but not used in clinical decision making | Aware and used in clinical decision making |
| --- | --- | --- | --- | --- |
|  | **International Journal of Medical and Health Science** |  |  |  |
| 501 | Centre of Evidence-based medicine (CEBM) | 1 | 2 | 3 |
| 502 | Google Scholar | 1 | 2 | 3 |
| 503 | PubMed journal | 1 | 2 | 3 |
| 504 | HINARI: Access to Research in Health program | 1 | 2 | 3 |
|  | **Databases** | 1 | 2 | 3 |
| 505 | British Nursing index | 1 | 2 | 3 |
| 506 | Cochrane library(systematic reviews ) | 1 | 2 | 3 |
| 507 | Cumulative Index to Nursing and Allied Health Literature (CINAHL) | 1 | 2 | 3 |
| 508 | Medline (Extensive Medical and Nursing database) | 1 | 2 | 3 |
|  | **Applications** |  |  |  |
| 509 | Medscape | 1 | 2 | 3 |
| 510 | Up-to-date | 1 | 2 | 3 |
| 511 | Medical Skills and Procedures | 1 | 2 | 3 |

**Section VI: Nurses Information searching skill for evidence-based practice**

| S.N | Items | Scales | | | | |
| --- | --- | --- | --- | --- | --- | --- |
|  |  | Very poor | Poor | Neutral | Good | Very good |
| 601 | The skill of using a computer and the internet to search for online information via database and/or search engines | 1 | 2 | 3 | 4 | 5 |
| 602 | Skill at browsing the internet, information searching and retrieving skills in the context of EBP | 1 | 2 | 3 | 4 | 5 |
| 603 | Skill at using search strategy(Index browsing (e.g. author, title, resource) Truncations/wildcards (e.g. ‘*’, ‘?’), Medical Subject Headings (MeSH), Search limits (e.g. publication date), Proximity operators (e.g. W/nn) | 1 | 2 | 3 | 4 | 5 |
| 604 | Skill of download and/or upload information through internet | 1 | 2 | 3 | 4 | 5 |
| 605 | When searching the best clinical evidence from electronic databases, your use of Boolean operators/connectors (“OR”, “AND”, “NOT” or “NEAR”). | 1 | 2 | 3 | 4 | 5 |
| 606 | Your foreign language skill to read and understand international best evidence | 1 | 2 | 3 | 4 | 5 |

|  | | **Section VII: Nurses attitude towards evidence-based practice** | | | | | |
| --- | --- | --- | --- | --- | --- | --- | --- |
| **S.N** | **Items** | | **Choices** | | | | |
|  |  |  | **Strongly**  **Disagree** | **Disagree** | **Neutral** | **Agree** | **Strongly**  **Agree** |
| 701 | Literature and research findings are useful in my day-to-day practice | | 1 | 2 | 3 | 4 | 5 |
| 702 | Application of Evidence-Based Practice is necessary for the practice related to my profession | | 1 | 2 | 3 | 4 | 5 |
| 703 | I need to increase the use of evidence in my daily practice. | | 1 | 2 | 3 | 4 | 5 |
| 704 | I am interested in learning or improving the skills necessary to incorporate Evidence-Based Practice into my practice. | | 1 | 2 | 3 | 4 | 5 |
| 705 | Evidence Based Practice improves the quality of patient care. | | 1 | 2 | 3 | 4 | 5 |
| 706 | My reimbursement rate will increase if I incorporate Evidence-Based Practice into my practice. | | 1 | 2 | 3 | 4 | 5 |
| 707 | Evidence-Based Practice helps me to make decisions about patient care. | | 1 | 2 | 3 | 4 | 5 |
| 708 | I believe my workload is not too high to keep up-to-date with all new evidence. | | 1 | 2 | 3 | 4 | 5 |
| 709 | I believe that critically assessing evidence is an important step in evidence-based practice | | 1 | 2 | 3 | 4 | 5 |
| 710 | I believe that I have access to the best resources needed to carry out evidence-based practices. | | 1 | 2 | 3 | 4 | 5 |

| **Section VIII: Self-Efficacy towards evidence-based practice**  **Instruction**: the following items describe activities that support and ensure evidence-based nursing practice. Rate how confident you are that you can do each activity listed using a scale labeled by a number from 0 to 100% | | | | |
| --- | --- | --- | --- | --- |
| **S.N** | **Items** | 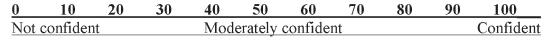 | | |
|  |  | **Not Confident** | **Moderately Confident** | **Confident** |
| 801 | Routinely ask answerable questions in my clinical practice | 1 | 2 | 3 |
| 802 | Locate resources in my department and institution to facilitate my understanding of research literature relevant to my nursing practice | 1 | 2 | 3 |
| 803 | Locate and review published research studies that have relevance to nursing interventions important to my practice | 1 | 2 | 3 |
| 804 | Routinely identify patient outcomes to target nursing interventions | 1 | 2 | 3 |
| 805 | Integrate the various sources of evidence and apply to my specialty and practice | 1 | 2 | 3 |
| 806 | Modify nursing interventions recommended for my patient based on characteristics of the specific unit in which I work | 1 | 2 | 3 |
| 807 | Routinely evaluate the research literature and other sources of evidence related to nursing interventions for my specialty population and practice | 1 | 2 | 3 |

**Section IX: Factors affecting evidence-based practice**

| **S.N** | **Questions** | **Choices** | **Skip** |
| --- | --- | --- | --- |
| 901 | Which of the following health information sources are available in your organization?  (More than one choice is possible) | 1. paper journals 2. Books 3. Relevant practice guidelines 4. Access to an online database 5. Training manuals 6. All of the above 7. None of the above 8. Others, specify___ |  |
| 902 | Is there access to computers loaded with different reference materials (like books, journals, guidelines)? | 1) Yes  2) No |  |
| 903 | Is there internet access in your workplace? | 1) Yes  2) No | If Q903 is No go to Q905 |
| 904 | Is there a high speed of the internet in your working area? | 1) Yes  2) No |  |
| 905 | Does your facility administration support the use of current research in your clinical practice? | 1) Yes  2) No |  |
| 906 | Have you received formal training in search strategies for finding research relevant to your practice? | 1) Yes  2) No |  |
| 907 | Do you have enough time to apply evidence-based practice? | 1) Yes  2) No |  |
| 908 | Do you have an awareness about the research and Evidence-Based Practice? | 1) Yes  2) No |  |
| 909 | Do you be able to read and understand materials in English or other languages | 1) Yes  2) No |  |
| 910 | Do you feel that you have enough authority to change patient care procedures? | 1) Yes  2) No |  |
| 911 | Are physicians cooperating with the EBP implementation? | 1) Yes  2) No | |
| 912 | Does the used statistical analysis are easily understandable? | 1) Yes  2) No | |
| 913 | Does literature reports have conflicting results with your clinical practice? | 1) Yes  2) No | |
| 914 | Are there sufficient resources (e.g. equipment, protocols, guidelines) in your facility to change practice | 1) Yes  2) No | |
| 915 | Rank your 3 greatest factors to the use of Evidence-Based Practice in your clinical practice. | □ Insufficient time  □ Lack of information resources  □ Lack of research skills  □ Poor ability to critically appraise the literature  □ Inability to apply research findings to individual patients with unique characteristics  □ Lack of understanding of statistical analysis  □ Lack of collective support among my colleagues in my facility | |
| 916 | In your opinion, what factors are important to be corrected for you to adopt Evidence-Based Practice? | 1. Give adequate training in searching strategies & EBP 2. Give protected time to conduct EBP 3. Access to a system for comprehensive literature searching 4. Mentoring by nurses who have adequate EBP experience 5. If others, specify__________ | |

**Thank you for your valuable response and patience!!!**

**Guiding questions for In-depth Interview English Version**

1. What is Evidence-Based Practice (EBP) to you? Do you know to implement Evidence-Based Practice (EBP) principles? Is there any implemented Evidence-Based Practice? If yes, what are those? Are those have a positive outcome? If so what are those outcomes?
2. Can you tell me about the implementation of Evidence-Based Practice in your setting? Does it have importance or not? If not, why? If yes, tell me them? What are the problems with the implementation of Evidence-Based Practice? Is that difficult or easy? If yes, how?
3. How can we improve the implementation of Evidence-Based Practice? Are you ready to improve the implementation of Evidence-Based Practice? If yes, How? If not why? Is your organization or leaders help in implementing Evidence-Based Practice? If yes how? If not, why? Are you helping your organization or leader to implement Evidence-Based Practice?
4. Have you read nursing researches based on your clinical problem? If yes are you try to share with others? If no why? If yes how do you share?

**የጥናቱ የአማርኛ መጠይቅ (Guiding questions for In-depth Interview Amharic Version)**

1. እድሜዎን ከዛም መረጃን ተመርኩዞ መስራት ማለት ለእርስዎ ምን ማለት እንደሆነ ቢነግሩን? በመረጃ ላይ የተመሠረተ የነርሲንግ አገልግሎት አሰራር መርሆዎችን ለመተግበር ዕውቀቱ አለዎት? መረጃን ተመርኩዞ የመስራት ትግበራ አለ? መልስዎ አዎ ከሆነ ፣ የትኞቹ ናቸው? አዎንታዊ ውጤት አላቸው? ከሆነስ ውጤቶች ምንድን ናቸው? ቢያብራሩልኝ?
2. ስለ በመረጃ ላይ የተመሠረተ የነርሲንግ አገልግሎት አሰራር አፈፃፀም ሊነግሩኝ ይችላሉ? አስፈላጊ ነው ወይስ አይደለም? ካልሆነ ለምን? አዎ ከሆነ ብትነግረኝ(ሪኝ)? በመረጃ ላይ የተመሠረተ አሰራር አፈፃፀም ላይ ችግሮች የትኞቹ ናቸው? ያስቸግራሉ ወይስ ቀላል ናቸው? አዎ ከሆነ ፣ እንዴት?
3. በመረጃ ላይ የተመሠረተ አሰራር አፈፃፀምን እንዴት ማሻሻል እንችላለን? በመረጃ ላይ የተመሠረተ አሰራር አፈፃፀምን ለማሻሻል ዝግጁ ነዎት? አዎ ከሆነ ፣ እንዴት? ካልሆነ ለምን? ሆስፒታሉ ወይም አመራሮች በመረጃ ላይ የተመሠረተ አሰራርን ለመተግበር እገዛ ያደርጋሉ? አዎ ከሆነ እንዴት? ካልሆነ ለምን? በመረጃ ላይ የተመሠረተ አሰራርን ተግባራዊ ለማድረግ ድርጅትዎን ወይም መሪዎን እየረዱ ነው?
4. በነርሲንግ አገልግሎት የአሰራር ችግርዎ ላይ ተመስርተው የነርሶች ምርምሮችን አንብበዋል? አዎ ከሆነ ለሌሎች ለማጋራትስ ይሞክራሉ? ካልሆነ ለምን?

**ጊዜዎትን ሰውተው ስለተባበሩኝ እጅግ አመሰግናለሁ::**
