# Supplementary material for: Evidence-based practice and its associated factors among point-of-care nurses working at the teaching and specialized hospitals of Northwest Ethiopia: A concurrent study
Source: PLoS One. 2022 May 5;17(5):e0267347. doi: 10.1371/journal.pone.0267347 (PMC9070954; doi:10.1371/journal.pone.0267347)
Supplement: S1 File — (DOCX) [file pone.0267347.s004.docx]

**የጥናቱ የአማርኛ መጠይቅ (Guiding questions for In-depth Interview Amharic Version)**

1. እድሜዎን ከዛም መረጃን ተመርኩዞ መስራት ማለት ለእርስዎ ምን ማለት እንደሆነ ቢነግሩን? በመረጃ ላይ የተመሠረተ የነርሲንግ አገልግሎት አሰራር መርሆዎችን ለመተግበር ዕውቀቱ አለዎት? መረጃን ተመርኩዞ የመስራት ትግበራ አለ? መልስዎ አዎ ከሆነ ፣ የትኞቹ ናቸው? አዎንታዊ ውጤት አላቸው? ከሆነስ ውጤቶች ምንድን ናቸው? ቢያብራሩልኝ?
2. ስለ በመረጃ ላይ የተመሠረተ የነርሲንግ አገልግሎት አሰራር አፈፃፀም ሊነግሩኝ ይችላሉ? አስፈላጊ ነው ወይስ አይደለም? ካልሆነ ለምን? አዎ ከሆነ ብትነግረኝ(ሪኝ)? በመረጃ ላይ የተመሠረተ አሰራር አፈፃፀም ላይ ችግሮች የትኞቹ ናቸው? ያስቸግራሉ ወይስ ቀላል ናቸው? አዎ ከሆነ ፣ እንዴት?
3. በመረጃ ላይ የተመሠረተ አሰራር አፈፃፀምን እንዴት ማሻሻል እንችላለን? በመረጃ ላይ የተመሠረተ አሰራር አፈፃፀምን ለማሻሻል ዝግጁ ነዎት? አዎ ከሆነ ፣ እንዴት? ካልሆነ ለምን? ሆስፒታሉ ወይም አመራሮች በመረጃ ላይ የተመሠረተ አሰራርን ለመተግበር እገዛ ያደርጋሉ? አዎ ከሆነ እንዴት? ካልሆነ ለምን? በመረጃ ላይ የተመሠረተ አሰራርን ተግባራዊ ለማድረግ ድርጅትዎን ወይም መሪዎን እየረዱ ነው?
4. በነርሲንግ አገልግሎት የአሰራር ችግርዎ ላይ ተመስርተው የነርሶች ምርምሮችን አንብበዋል? አዎ ከሆነ ለሌሎች ለማጋራትስ ይሞክራሉ? ካልሆነ ለምን?

**ጊዜዎትን ሰውተው ስለተባበሩኝ እጅግ አመሰግናለሁ::**
