# Supplementary material for: Evidence-based practice and its associated factors among point-of-care nurses working at the teaching and specialized hospitals of Northwest Ethiopia: A concurrent study
Source: PLoS One. 2022 May 5;17(5):e0267347. doi: 10.1371/journal.pone.0267347 (PMC9070954; doi:10.1371/journal.pone.0267347)
Supplement: Attachment — Submitted filename: PONE-D-21-35844.pdf [file pone.0267347.s006.pdf]

PLOS ONE

Evidence-Based Practice and its Associated Factors among Point-of-care Nurses working at the Teaching and Specialized Hospitals of Northwest Ethiopia: A Concurrent Study

--Manuscript Draft--

**Manuscript Number:**	PONE-D-21-35844
**Article Type:**	Research Article
**Full Title:**	Evidence-Based Practice and its Associated Factors among Point-of-care Nurses working at the Teaching and Specialized Hospitals of Northwest Ethiopia: A Concurrent Study
**Short Title:**	EBP and its Associated Factors among Nurses at Teaching Hospitals of Northwest Ethiopia: A Concurrent Study
**Corresponding Author:**	abebe birhanu degu, MPH<br>UNIVERSITY OF GONDAR<br>GONDAR, Amhara ETHIOPIA
**Keywords:**	Evidence-Based Practice; Nursing Practice; Ethiopia
**Abstract:**	Background<br><br>Evidence-based practice (EBP) is the application of the best scientific evidence for clinical decision making in professional patient care. Most of the time, nursing care practice in Ethiopia is based on experience, tradition, intuition, common sense, and untested theories. Clinical practicing based on evidence could improve the quality of healthcare services, making it cost-effective, and improving clinical outcomes.<br><br>Methods<br><br>An institutional-based concurrent study design method of quantitative and qualitative was conducted from Feb 30 to Apr 20, 2020. Systematic random sampling and purposive sampling techniques were used to select the study participants for the quantitative and qualitative, respectively. Quantitative data were collected using a pretested, structured, and self-administered questionnaire, and the qualitative data were collected by an in-depth interview guiding questions.<br><br>Results<br><br>EBP was found to be significantly associated with educational level (AOR=2.15, CI (1.15-4.02)), administrative support for EBP (AOR=1.89, CI (1.22-2.91)), attitude towards EBP (AOR=1.80, CI (1.24-2.62)), and preference of available information sources (AOR: 2.32, CI: (1.58-3.39)). Four main themes arose by conventional content data analysis: (1) advantages of the application of EBP (2) barriers for the implementation of EBP (3) Enabling factors for EBP and (4) Sharing of evidence.<br><br>Conclusion<br><br>This study concluded that a small number of nurses applied EBP to a high level. There was a positive association between educational level, attitude towards EBP, administrative support, the availability of information resources with implementation of EBP and it was confirmed by the qualitative study. There has to be an intervention program to facilitate the implementation of evidence in nursing practice by the stakeholders to improve and increase the efficacy of practicing EBP.
**Order of Authors:**	abebe birhanu degu, MPH
	Tesfahun Melese Yilma, PHD
	Miftah Abdella Beshir, MPH
	anushia inthiran, PHD

Powered by Editorial Manager® and ProduXion Manager® from Aries Systems Corporation

Additional Information:	
**Question**	**Response**
**Financial Disclosure**<br><br>Enter a financial disclosure statement that describes the sources of funding for the work included in this submission. Review the submission guidelines for detailed requirements. View published research articles from *PLOS ONE* for specific examples.<br><br>This statement is required for submission and **will appear in the published article** if the submission is accepted. Please make sure it is accurate.<br><br>**Unfunded studies**<br>Enter: *The author(s) received no specific funding for this work.*<br><br>**Funded studies**<br>Enter a statement with the following details:<br>• Initials of the authors who received each award<br>• Grant numbers awarded to each author<br>• The full name of each funder<br>• URL of each funder website<br>• Did the sponsors or funders play any role in the study design, data collection and analysis, decision to publish, or preparation of the manuscript?<br>• **NO** - Include this sentence at the end of your statement: *The funders had no role in study design, data collection and analysis, decision to publish, or preparation of the manuscript.*<br>• **YES** - Specify the role(s) played.<br><br>* typeset	The author(s) received no specific funding for this work
**Competing Interests**<br><br>Use the instructions below to enter a competing interest statement for this submission. On behalf of all authors, disclose any competing interests that could be perceived to bias this work—acknowledging all financial support and any other relevant financial or non-financial competing interests.	The authors have declared that no competing interests exist

This statement is required for submission and **will appear in the published article** if the submission is accepted. Please make sure it is accurate and that any funding sources listed in your Funding Information later in the submission form are also declared in your Financial Disclosure statement.<br><br>View published research articles from *PLOS ONE* for specific examples.<br><br>**NO authors have competing interests**<br><br>Enter: *The authors have declared that no competing interests exist.*<br><br>**Authors with competing interests**<br><br>Enter competing interest details beginning with this statement:<br><br>*I have read the journal's policy and the authors of this manuscript have the following competing interests: [insert competing interests here]*<br><br>* typeset	
**Ethics Statement**<br><br>Enter an ethics statement for this submission. This statement is required if the study involved:<br><br>• Human participants<br>• Human specimens or tissue<br>• Vertebrate animals or cephalopods<br>• Vertebrate embryos or tissues<br>• Field research<br><br>Write "N/A" if the submission does not require an ethics statement.<br><br>General guidance is provided below. Consult the submission guidelines for detailed instructions. **Make sure that all information entered here is included in the**	Ethical clearance was obtained from the Institutional Review Board of the University of Gondar. Official letters of support were presented to the University of Gondar and Tibebe Gion teaching specialized hospitals and the intents and the grandness of the subject were explained & verbal informed consent was obtained from each participant.

**Methods section of the manuscript.**<br><br>**Format for specific study types**<br><br>**Human Subject Research (involving human participants and/or tissue)**<br>• Give the name of the institutional review board or ethics committee that approved the study<br>• Include the approval number and/or a statement indicating approval of this research<br>• Indicate the form of consent obtained (written/oral) or the reason that consent was not obtained (e.g. the data were analyzed anonymously)<br><br>**Animal Research (involving vertebrate animals, embryos or tissues)**<br>• Provide the name of the Institutional Animal Care and Use Committee (IACUC) or other relevant ethics board that reviewed the study protocol, and indicate whether they approved this research or granted a formal waiver of ethical approval<br>• Include an approval number if one was obtained<br>• If the study involved *non-human primates*, add *additional details* about animal welfare and steps taken to ameliorate suffering<br>• If anesthesia, euthanasia, or any kind of animal sacrifice is part of the study, include briefly which substances and/or methods were applied<br><br>**Field Research**<br><br>Include the following details if this study involves the collection of plant, animal, or other materials from a natural setting:<br>• Field permit number<br>• Name of the institution or relevant body that granted permission	
**Data Availability**<br><br>Authors are required to make all data underlying the findings described fully available, without restriction, and from the time of publication. PLOS allows rare exceptions to address legal and ethical concerns. See the PLOS Data Policy and FAQ for detailed information.	Yes - all data are fully available without restriction

Powered by Editorial Manager® and ProduXion Manager® from Aries Systems Corporation

A Data Availability Statement describing where the data can be found is required at submission. Your answers to this question constitute the Data Availability Statement and **will be published in the article**, if accepted.<br><br>**Important:** Stating 'data available on request from the author' is not sufficient. If your data are only available upon request, select 'No' for the first question and explain your exceptional situation in the text box.<br><br>Do the authors confirm that all data underlying the findings described in their manuscript are fully available without restriction?	
**Describe where the data may be found in full sentences. If you are copying our sample text, replace any instances of XXX with the appropriate details.**<br><br>• If the data are **held or will be held in a public repository**, include URLs, accession numbers or DOIs. If this information will only be available after acceptance, indicate this by ticking the box below. For example: *All XXX files are available from the XXX database (accession number(s) XXX, XXX.*).<br>• If the data are all contained **within the manuscript and/or Supporting Information files**, enter the following: *All relevant data are within the manuscript and its Supporting Information files.*<br>• If neither of these applies but you are able to provide **details of access elsewhere**, with or without limitations, please do so. For example:<br><br>*Data cannot be shared publicly because of [XXX]. Data are available from the XXX Institutional Data Access / Ethics Committee (contact via XXX) for researchers who meet the criteria for access to confidential data.*<br><br>*The data underlying the results*	all data will be available at the corresponding author

presented in the study are available from (include the name of the third party and contact information or URL).<br>• This text is appropriate if the data are owned by a third party and authors do not have permission to share the data.<br><br>* typeset	
Additional data availability information:	Tick here if the URLs/accession numbers/DOIs will be available only after acceptance of the manuscript for publication so that we can ensure their inclusion before publication.

Powered by Editorial Manager® and ProduXion Manager® from Aries Systems Corporation

Manuscript
Click here to access/download;Manuscript;Abebebirhanu_Manuscript.docx

Evidence-Based Practice and its Associated Factors among Point-of-care Nurses working at the Teaching and Specialized Hospitals of Northwest Ethiopia: A Concurrent Study

Abebe Birhanu Degu*[1], Tesfahun Melese Yilma[1], Miftah Abdella Beshir[1], Anushia Inthiran[2]

***correspondence:** abebirhanu21@gmail.com

[1] Department of Health Informatics, College of Medicine and Health Sciences, University of Gondar, Gondar, Ethiopia

[2] Department of Accounting and Information Systems, College of Business and Law, University of Canterbury, Meremere, New Zealand

Full list of author information is available at the end of the article

Abstract

Background: Evidence-based practice (EBP) is the application of the best scientific evidence for clinical decision making in professional patient care. Most of the time, nursing care practice in Ethiopia is based on experience, tradition, intuition, common sense, and untested theories. Clinical practicing based on evidence could improve the quality of healthcare services, making it cost-effective, and improving clinical outcomes.

Methods: An institutional-based concurrent study design method of quantitative and qualitative was conducted from Feb 30 to Apr 20, 2020. Systematic random sampling and purposive sampling techniques were used to select the study participants for the quantitative and qualitative, respectively. Quantitative data were collected using a pretested, structured, and self-administered questionnaire, and the qualitative data were collected by an in-depth interview guiding questions.

Results: EBP was found to be significantly associated with educational level (AOR=2.15, CI (1.15-4.02)), administrative support for EBP (AOR=1.89, CI (1.22-2.91)), attitude towards EBP (AOR=1.80, CI (1.24-2.62)), and preference of available information sources (AOR: 2.32, CI: (1.58-3.39)). Four main themes arose by conventional content data analysis: (1) advantages of the application of EBP (2) barriers for the implementation of EBP (3) Enabling factors for EBP and (4) Sharing of evidence.

Conclusion: This study concluded that a small number of nurses applied EBP to a high level. There was a positive association [illegible]ween educational level, attitude towards EBP, administrative support, the availability of information resources with implementation of EBP and it was confirmed by the qualitative study. There has to be an intervention program to facilitate the implementation of evidence in nursing practice by the stakeholders to improve and increase the efficacy of practicing EBP.

Keywords: *Evidence-Based Practice, Nursing Practice, Ethiopia*

Background

Evidence-based practice (EBP) is the application of the best scientific evidence in clinical decision-making by integrating clinical experience and incorporating patient values and preferences, in the practice of professional patient care (1). It is about making decisions by using the best available evidence from multiple sources in a conscientious, explicit, and judicious manner (2).

Evidence is information based on historical or scientific evaluation of practices that can be accessible to decision-makers in the health care system. The types of evidence considered acceptable include: experimental (randomized clinical trials, meta-analyses, and analytic studies); non-experimental (quasi-experimental, observational); expert opinion and historical or experiential (3).

Nursing is a science that is essential to draw knowledge from research findings. Scientific research is the standard by which knowledge is derived from science. Practice in nursing is the source of research questions, while research is the basis of current practice. Therefore, practice and research exist with each other in a circular continuum (4).

Currently, EBP, which is the use of theoretical research-based findings together with reliable forms of evidence in clinical decision-making, is essential to nursing practice to promote optimal patient outcomes by incorporating research findings, the experience of clinicians, and patient preferences (5).

EBP has been recognized as the gold standard for the delivery of compassionate and safe care as well as promoting excellence in nursing care. Despite the exploding availability of healthcare information and the ongoing pressure by authorities, the application of research-based evidence in nursing practice is limited; the care of the patient was influenced by the experiences and opinions of those involved in providing treatment. Studies consistently show that interventions have failed to be effective and cost-effective in worldwide (6).

EBP is not widely embraced in low- and middle-income countries. EBP methods are a relatively new and often overwhelming challenge for many healthcare organizations. For

example, in Africa such as South Africa, Ethiopia, Kenya, Nigeria, Egypt, Botswana, Burundi, and Malawi EBP is emphasized and promoted for nurses (**7-10**). Yet, the development of EBP in nursing practice is in its infancy stage. For example, a recent study from Nigeria reported that EBP is not widely developed in the context of the country's health care system (**7**). EBP in Africa is remaining challenging. One reason for this challenge is Africa lags in research because limited studies are describing the state of the art of EBP and the lack of funding from the government (**10**). Another possible reason is due to limited resource which makes it almost impossible for health professionals to work with vulnerable communities to access information in low-income settings (**11**).

Nurses may not have the skill or expertise on how to obtain the research evidence from the literature or how to apply the evidence, which makes it cost-ineffective, none improving clinical outcomes, obstacles towards transferring research into clinical practice, and challenging the adoption process (**12**). Such a situation produces medical errors such as miss diagnosis, wrong treatment, increase in multidrug resistance, sever iatrogenic injuries, and unexpected patient death which are common in today's health care services (**13, 14**).

The nursing staff is the largest health professional group in all health care sectors who are working in the direct care of patients, assessing patients' needs, and making decisions on nursing interventions that will stay for a long period (**15**). However, at all levels of the health systems, there is little culture or tradition of conducting, trusting, or using EBP by nurses (**16**).

Even though several studies have attempted to analyze EBP from different perspectives, the detailed factors are not analyzed, especially in developing countries like Ethiopia and also in the study area while professional development and information communication technologies (ICT) are becoming more advanced. If nurses do perform EBP that would be beneficial for continuous education, continuous learning, improved knowledge, and overall be able to increase the quality of care given to patients. Additionally, it is not known how much health-related research is being utilized to improve healthcare practice to ensure high-quality healthcare delivery and improve standards and quality of life. Hence, this study aimed to assess Evidence-Based Practice and its associated factors among nurses working at the study settings.

Methods

Research Design and Setting

An institution-based concurrent study design using quantitative and qualitative methods was conducted from 30 Feb to 20 Apr 2020. This research was carried out in the two governmental teaching and specialized hospitals of the Amhara Regional State, Northwest Ethiopia (the University of Gondar and Tibebe Gion), serving 3.5–5 million people each. The hospitals are the only teaching and specialized hospitals in the Amhara region. They are selected because:

- They are training centers for professional development as in-service and out-service for health professionals in addition to undergraduate and postgraduate medical students.
- They have a reputation for research excellence since they are staffed with the most senior specialists who manage patients with complex illness and are exposed to highly specialized care with well resourced diagnostic and therapeutic facilities as a larger referral center in the region.
- They have strategic goals to produce evidence-based health care professionals with a culture of continuous professional development and improved patient care.
- They are serving as a practical educational site for students and healthcare providers in addition to their daily patient care so nurses are expected to implement EBP.

Research Participants

Inclusion criteria: This study was included all nurses having a Bachelor of Science (BSc) and above in nursing who are working at the study settings.

Exclusion criteria: This study was excluded nurses who were on leave (annual, maternal, and/or sick leave), having less than six-month of work experience, nurses who were on postgraduate programs, and nurses of academic staffs since postgraduate program followers/students were not eligible during the data collection period and

Nurses involved in academic staff were believed to have better EBP experience in their academic activities (17), who skewed the results of the study.

Sample Size Determination

The sample size was calculated using both the single and double population proportion formula.
Single population proportion formula:

$$n = \frac{(z_{\alpha/2})^2 * p\,(1-p)}{(d)^2}$$

Where, z = 95% confidence level, d = 5% margin of error, and 51.8% proportion (p) of EBP from previous study (**16**). Assuming a 10% non-response rate, the total sample size became 423.

Double population proportion formula:

We have calculated the sample size for each factor variables from the previous study (**16**). Power =80%, confidence level 95%, OR 1:1 (Table: 1 see Additional file 1)

Then taken the highest sample size i.e. 482 and assumed the non-response rate of 10 % (482) =48 the final sample size was determined to be 482+48=**530**.

Sampling Procedures

The study participants were selected proportionally from the two hospitals. The lists of nurses in each hospital were used as a sampling frame for identifying possible study participants in the sample. All nurses working at the same hospital were assumed to be homogeneous for EBP. Participants of the study were then randomly selected using record identification numbers taken from the sampling frame. The lottery method was used to randomly pick nurses as study participants (Fig 1 see Additional file 2).

Among 530 candidate study participants a total of 507 BSc and above nurses, who returned the questionnaire were taken from 10 administrative nurses, 34 nurses' team leaders, whereas 463 as staff nurses by simple random sampling.

For an in-depth interview 12 key informants were taken by convenience till information saturation has been made. The key informant selection criteria were both professional and practical experience (clinical staff and/or management). The first selection criterion was to work at least during the last 6 months in the hospital. Upon meeting these criteria, nurses were divided into the following groups: 1. Administrative nurses group (G ADM) 2. Team leader nurses group (G TEAM) and 3. Clinical Staff nurses: additionally, these nurses were subdivided into three groups:

3.1. Less than 5 years of experience in the hospital (G STA<5),

3.2. Between 5 and 10 years of experience in the hospital (G STA5–10),

3.3. More than 10 years of experience in the hospital (G STA > 10).

Data Collection Tools and Procedures

Data were collected using a pretested and structured self-administered questionnaire which was mostly adapted from various studies conducted in Ethiopia previously **(10, 16, 18**) and some of them were adopted from various studies (**19, 20**) (see Additional file 5). The questionnaire was prepared in English as the study subjects were BSc and above nurse professionals who are believed to be proficient in English. For the in-depth interview, the guiding questions were prepared and translated into the local language (Amharic) for its simplicity, validity, and clarity.

Moreover, the questionnaire was pretested among 5% of the sample size at Felege Hiwot Referral Hospital, and questionnaire reduction, jargon words elimination, grammar correction, etc. were done. The reliability of the tool for each subscale was checked using Cronbach's alpha reliability test (0.91), which indicated sufficient reliability of parameters in the questionnaire.

Quantitative data were collected from nurses by one BSc nurses and one health informatics profession through distributing structured self-administered questioner for the nurses after explaining the purpose and technique of filling the questionnaire. The Principal Investigator (PI) has done continuous monitoring and supervision throughout the data collection period.

Qualitative data was collected by the PI from key informants. Key informants took for an in-depth interview based on purposive convenience sampling. Every in-depth interview had been taken 30-35 minutes. Notes and audio recorders have been used to record information from key informants.

The questionnaire of the quantitative study were consisted of eight sections: about EBP, Knowledge, Preferences/Use of available information sources, Awareness about Electronic Information Sources, Information searching skill, Nurses Self-Efficacy**,** Attitude, and Factors associated with evidence-based practice. Each section had its own number of subscale having greater than seven questions made by different researchers which measures it.

In total, seven self-reported questions were asked to test EBP. Each question had a response scale of four points (never, monthly, weekly, and daily). These categories were marked from 1 to 4 as numerical values (Never '1', Monthly '2', Weekly '3', Daily '4'). Respondent, therefore, had a total score of EBP (from a minimum of 7 to a possible maximum of 28). The average of each question can be calculated as (1+2+3+4)/4= 2.5. Answering above 2.5 was considered as desirable responses to the EBP questions. EBP was assessed by using the average score of the seven questions. Hence, 2.5x7=17.5 is used as the cut-off point. Respondents having a total EBP score above the cut of point 17.5 were considered as having 'Good EBP' while those below were labeled as having 'Poor EBP'.

In total, 10 questions showing the extent of EBP knowledge, each of which had a Yes, No, and I don't know (IDK) answer, was asked. Yes, answers are coded as '1', and 'no', and I don't know (IDK) as '0'. A cumulative EBP knowledge score (from at least 0 up to a maximum of 10) was determined for all respondents. After checking for normal distribution the mean knowledge score was calculated to test the knowledge score using Shapiro–Wilk test (21) (W= 0.982 and p= 0.11) and a visual inspection of their histogram, normal Q-Q plots, and box plots showed that knowledge scores were approximately normally distributed with a skewness = -0.167 (SE=0.218), kurtosis of -0.247(SE=0.433) and mean = 6 (SD=±1.73). Respondents having knowledge score above the mean value considered as 'good knowledge' unless as 'poor knowledge'.

The level of attitude towards EBP was determined in ten questions. Every question of attitude had a Likert scale of five points (strongly agreed coded as 5, agreed coded as 4, neutral coded as 3, disagreed coded as 2, and strongly disagreed coded as 1). Each respondent had a cumulative attitude score of at least 10 points to a possible maximum of 50 points. The attitude towards an EBP score was tested for normal distribution using the Shapiro Wilk test (W= 0.983, p= 0.589) and a visual inspection of their histogram, normal Q-Q plots, and box plots showed that attitude scores were approximately normally distributed with a skewness=0.015 (SE=0.316), kurtosis of -0.206 (SE=0.623) and mean=36 (SD=±3.921). Respondents with an attitude above the mean score towards evidence-based practice were classified as 'favorable attitude' and those below were categorized as 'unfavorable attitude'.

In total, six questions to measure the level of respondents' skill towards Information Searching skills (skill in finding, reviewing, and using different sources of evidence) had a Likert scale of five points (very good coded as 5, Good coded as 4, neither good nor poor coded as 3, poor coded as 2, and very poor coded as 1). For all respondents, a total EBP skill score (from a minimum of 6 up to a possible maximum of 30) was determined. The mean skill score was calculated after checking for normal distribution to test the skill score using the Shapiro–Wilk test (21) (W= 0.971, p= 0.09) and a visual inspection of their histogram, normal Q-Q plots, and box plots showed that skill scores were approximately normally distributed with a skewness= -0.124 (SE= -0.279), kurtosis of -0.206 (SE= 0.552) and mean= 19 (SD=2.968) and label respondents having skill scores above the mean value as 'good skill' unless as 'poor skill'.

In total, seven questions were provided to measure the use of available information sources with a Likert scale of five points (never "1", rarely "2", sometimes "3", often "4" and always "5"). The mean use score was calculated after checking for normal distribution to test the use score using the Shapiro–Wilk test (21) (W= 0.945, p= 0.79) and a visual inspection of their histogram, normal Q-Q plots, and box plots showed that preference scores were approximately normally distributed with a skewness= -0.249 (SE= 0.398), kurtosis of 0.335 (SE= 0.495) and mean= 12.8 (SD=0.474) and label

respondents having use scores above the mean value as ‘preferred available information source’ unless as ‘not preferred’.

Eleven questions were provided to measure awareness of electronic information sources with a Likert scale of three points (unaware “1”, aware but not used in clinical decision making “2”, aware and used “3”). The mean awareness score was calculated after checking for normal distribution to test the awareness score using the Shapiro–Wilk test (21) (W= 0.969, p= 0.197) and a visual inspection of their histogram, normal Q-Q plots, and box plots showed that awareness scores were approximately normally distributed with a skewness= -0.004 (SE=0.333), kurtosis of -0.368 (SE= 0.656) and mean= 14 (SD=0.487) and label respondents having awareness scores above the mean value as ‘aware electronic information source’ unless as ‘not aware’.

Seven questions were provided to measure the self-efficacy of nurses with a Likert scale of three points (not confident “1”, moderately confident “2” and confident “3”). The mean self-efficacy score was calculated after checking for normal distribution to test the self-efficacy score using the Shapiro–Wilk test (21) (W= 0.981, p= 0.258) and a visual inspection of their histogram, normal Q-Q plots, and box plots showed that preference scores were approximately normally distributed with a skewness= 0.172 (SE= 0.267), kurtosis of -0.302 (SE= 0.529) and mean= 14.72 (SD=0.307) and label respondents having self-efficacy scores above the mean value as ‘confident’ unless as ‘not confident’.

Data Management and Analysis

First, the data were coded and entered into Epi-data version 4.6.0 (Epi-Data Entry is used for simple or programmed data entry and data documentation. It handles simple forms or related systems Optimized documentation and error detection features) and exported to SPSS version 20 for analysis. Data analysis includes descriptive statistics to describe the demographic characteristics of the participants, and the data were described using text, tables, and graphs. A Chi-square test was done for all categorical independent variables. Variance inflation factor (VIF) was used to check multi-co linearity. Binary logistic regression was used to determine the association between the outcome variables and predictors. Statistical tests at 95% CI and P-value $\leq$ 0.05were

made. Variables having a p-value of up to 0.2 in the bi-variable analysis were selected to fit the model in the multivariable analysis. Finally, a P-value $\leq$ 0.05 in the multivariable model of logistic regression was used to select variables that had a statistically significant association with EBP. (See Additional file 3 and 4)

The qualitative data was taken by Amharic and then transcribed and interpreted in English. A thematic analysis was carried out through an inductive coding process. The researcher carried out the coding procedure to ensure the rigorous and credible of the analysis. Through this triangulation process, in a first reading of the data, the researcher identified emerging topics and patterns and drew up a list of possible topics that were then used to set up the codes based on Lundman and Graneheim data analysis approach. Data reduction was carried out by matching codes and setting relationships and analogies. The qualitative data analysis was assisted by the Open code software.

Trustworthiness was determined by using a technique of prolonged engagement with participants, persistent observation, peer-debriefing and member-checking. Evidence was also presented by iterative questioning of the data, and returning to examine it several times.

Ethical Consideration

Ethical clearance was obtained from the Institutional Review Board of the University of Gondar. Official letters of support were presented to the study settings and the intents and the grandness of the subject were explained and verbal informed consent was obtained from each participant. Participants were informed that all data obtained from them are maintained confidentially and anonymously by using codes instead of personal identifiers.

Results

Socio-Demographic Characteristics

A total of 530 questionnaires were distributed to the study participants with 507 of the questionnaires being returned (response rate of 95.7%). Of the returned questionnaires, 340 (67.1%) and 167 (32.9%) were from the University of Gondar and Tibebe Gion teaching specialized hospitals respectively. The respondents were 273 (53.8%) female and 234 (46.2%) male, 463 (91.3%) were staff nurses, and 34 (6.7%) were team lead nurses and 10 (2.0%) administrative nurses. Of the 507 study subjects, the mean age was 29.71(SD±4.42). Most of the subjects in the study were BSc, which accounts for 447 (88.2%), only 128 (25.2%) were got training related to EBP from 68 (13.4%) undergraduate and 60 (11.8%) postgraduate. The detailed information is provided in Table 2. (**Table: 2** See Additional file 1**)**

Evidence-Based Practice in the study area

Of the total 507 nurses, 239 (47%) [95% CI of 43% to 51%] had incorporated EBP into their clinical practice. Their practice rate was assessed with three options (sometimes / monthly, usually / weekly, and always / daily) by self-reporting. Just 160(32%), 216(43%), and 76 (15%) apply EBP monthly, weekly, and always or regularly in their clinical practice respectively (Fig: 2 See Additional file 2).

Knowledge about evidence-based practice

Once asked how familiar the respondents were with the concept, about 441 (87%) were familiar with the concept of EBP with a mean knowledge of 6 (SD±12.9). When the respondents asked questions about research terms about 217 (42.8%) of respondents scored low, and about 290 (57.2%) of respondents scored high with 58% [95% CI of 56.2% to 59.2%] of overall knowledge.

Preferences/Use of available information sources for Evidence-Based Practice

We found that human information sources (sharing with colleagues), using medical applications and policy and guidelines were used more frequently by them. It was

worthy of notice that the use of electronic information sources (medical journals and databases) among nurses was the least common (Fig: 3 See Additional file 2).

Awareness about Electronic Information Sources for Evidence-Based Practice

The majority of nurses have relatively aware and used medical applications such as Medscape than the different medical journals and databases of electronic information sources as seen in (Fig: 4 See Additional file 2).

Information searching skill for Evidence-Based Practice

Most nurses (60%) are unlikely to skim down or quickly browse resources. There are some problems, such as lack of information technology skills, access to research articles, lack of references in the native language that most research is published in a foreign language.

Even though participants had overall information searching skill of 64%, It was worth remembering that several nurses have revealed that they are not comfortable with Boolean and proximity operators (54%) (Fig: 5 See Additional file 2).

Nurses Self-Efficacy in Evidence-Based Practice

The majorities (71%) of nurses have no confidence but, only 5% of nurses have confidence in their decision making of day to day clinical practice (Fig: 6 See Additional file 2).

Attitude towards evidence-based practice

Respondents had a mean attitude score of 36 (SD±3.92), however, Fifty-five percent of respondents had a favorable attitude towards EBP. Based on the findings, 71% of respondents agreed that research and literature findings are useful in day-to-day practice, 58.5% of respondents agreed that EBP improves the quality of healthcare while 23.1% agreed that their reimbursement rate would increase if they incorporated EBP into their practice (Fig:7 See Additional file 2).

Factors associated with evidence-based practice

When asked about barriers that impeded their EBP, 52% of the participant reported that there were barriers to EBP implementation. The identified barriers includes: availability of information sources 16%(n=41), computer access 89%(n=235), internet access 89.7%(n=237), internet speed 53%(n=140), administrative support 74%(n=195), search strategy and EBP training 80%(n=211), enough time for practice 56%(n=148), awareness about EBP 58%(n=153), understanding literatures in foreign language 28%(n=74), authority to change 54%(n=143), physicians cooperation 55%(n=145), understanding statistical terms 54%(n=143), literatures have conflicting report 66%(n=174) and lack of resources 76%(n=201). Some respondents identified more than one barrier.

Qualitative result analysis

For an in-depth interview 12 BSc and above (7 staffs, 3 team leaders and 2 administrative) nurse key informants were selected purposively from 113 BSc and above nurses who were not selected for quantitative until the data was saturated.

Four main themes arose by conventional content data analysis: (1) advantages of the application of EBP (2) barriers for the implementation of EBP (3) Enabling factors for EBP (4) Sharing of evidence

1. **Advantages on the application of EBP**

This theme addressed what participants perceived as a constructive outcome when relying on data for their research and clinical practice, and that EBP decreases the uncertainty in clinical practice.

“I think we all operate in the same way that we have the best healthcare.” (G ADN) (M1)

They also believed that evidence-based healthcare provided a higher quality of care for patients and made the most of the resources and time available:

“I think you'll give more appropriate treatment to patients if you base yourself on the facts. For fewer resources, you can produce better outcomes.”(G TEAM) (M1)

Participants claimed that scientifically based clinical practice improves professional image and gives legal support.

“I believe that it makes the profession more valuable and reinforces it. Then, it is a back-up when you say. Well, we’re working on a scientific method.” (G TEAM) (M2)

They said that the EBP is encouraging and that it is both a challenge and motivation.

“Yes, I think so, too, when you see people doing things, you feel like doing things, too. When you go to another place where people are settled, they don't care about anything, and they just go to meet their work schedule...” (G STA >10) (F1)

“I would love to base the evidence on all my work. I will be happy if I could investigate at work. . . .” (G STA 5–10) (F2)

EBP leads to an awareness of what it means to be a nurse and what its professional responsibility is. Also, EBP entrusts the patient with a professional commitment.

“Yet, where do our shortcomings lie? We have to know them and know when we are doing our job properly...” (G STA 5- 10) (M1)

“Then, I have to do research, I have to answer this person who is coming and telling me something about Tuberculosis. . . .” (G TEAM) (M1)

2. **Barriers for the application of EBP**

Obstacles to applying EBP have arisen more often than the gains from its implementation. One participant noted the lack of incentives to encourage and recognize EBP, and stressed the importance of institutional support and motivation:

“In a way, I believe that it should be the responsibility of nursing management to motivate, encourage, and generate interest in nurses, and I do not know-how. I think training needs to come first and then encouragement.” (G STA 5–10) (F2)

“We clinical staff need our higher administrator's support in conducting clinical research because we know our problem more than academic staff, so if we have the opportunity to investigate our clinical problem, we can integrate research evidence into our everyday practices.” (G STA 5–10) (M2)

Nurses do not agree that variables such as finances, the type of patient or professional characteristics are taken into account when designing protocols or clinical practice guidelines:

"To start the protocol process, do we take into account the needs of each population group in which we work? This doesn't seem so, as it takes them into account when applying. . ." (G STA >10) (M3)

"Training center has to be established to give training for us on how to integrate research evidence in our practices and to develop guidelines for practice, thus serving as a source for guiding our health interventions" (G STA<5) (F3)

Participants found that various health practitioners dispute the clinical procedure. They mentioned the burden the hospital specialists placed on them, the patients, their colleagues, and the lack of written scientific documentation:

"The patients who push and tell you another nurse healed them the previous day and they did so differently and they are the ones who force you to do so. . ." (G STA >10) (F1)

"Throughout obtaining information and knowledge-based preparation are not provided and there is a lack of understanding and provision that the practitioner can work through the facilitator throughout developing a documentary environment." (G STA 5-10) (F4)

Participants were also very tired and frustrated with institutional help in their efforts to bring about sounder hospital improvements:

"We have just attended a conference, haven't we? Obscure things have we known, have we not? Then you send a session and say, look, it is explained here, not proof-based. The next day, you find out that they continue to do the same. It doesn't matter. . ." (G STA 5- 10) (M1)

Concerning the obstacles, doubts about new evidence and resistance to change were the most common problems mentioned. In all interviewees, weak arguments were presented as excuses for not applying EBPs, such as its complexity, and the lack of appropriate clinical resources and studies.

“Now that we are trying to apply EBP, yes, it is very complicated to be wondering all the time if you are doing it correctly.” (G STA 5–10)(M2)

“… No hospital library, revised rules, inspiration, adequate training, and computers to enable nurses to upgrade.” (G STA 5–10) (F2)

Participants also doubted the findings should be used in clinical practice:

“Instead, I believe the scientific method is very good, offering the career and all, but I do not think we need to become robots simply because it has been proved.” (G STA >10) (M3)

Participants agreed that, even though a theoretical foundation supports the new approach, it is very difficult for them to alter norms already developed. In every interview, this segment was addressed thoroughly, particularly in the interview with less experienced staff:

“It is difficult to change the first problem you face. People say to you: No, I always did so, so it works well.” (G STA 5–10)(M2)

They also mentioned an inadequate knowledge of EBP methodology:

“The first challenge is adapting, then, and preparing so that I can do it, and then, it's doubt, what if I do something wrong? What if I can't do that?” (G ADM) (M1)

3. Enabling factors for EBP

This theme included what participants regarded as an EBP facilitator.

“even if it is not enough sometimes there is training for some nurses, makes the Internet accessible to all professionals, training on information sources and methods of use, due to inadequate training and inadequacy of authority to change procedure of care, the nurses are unable to provide services based on a patient's informed choice.” (G STA 5–10) (F2)

“Training center has to be established to give training for us on how to integrate research evidence in our practices and to develop guidelines for practice, thus serving as a source for guiding our health interventions” (G STA<5) (F3)

4. Sharing of evidence

Concerning how new evidence is shared, all participants agreed that they do not receive formal working time during which they were taught by non-hospital experts, as well as by nurses from the hospital, who preferred to participate in the work:

"*In the sessions, we try to share it. For example, one of our colleagues has held an injection workshop because he knows that*." (G STA5-10) (F4)

"For if you say that I know nothing about it and that someone comes and does it, it means that you don't matter what you do." (G STA >10) (M3)

In situations and contexts not specifically designed to share scientific data, EBP has occasionally been promoted.

"It's usually in the tea break many times when we say: remember what we talked about a few days ago? Do you remember? That's a scientific paper I've found. . ." (G TEAM) (M1)

Participants further discussed variations in clinical quality and quantity and other methods of EBP sharing between hospitals.

"The difference is so vast that I have been working in several health centers and hospitals" (G STA>10) (F1)

Discussion

The purpose of this study was to assess Evidence-Based Practice and its associated factors among Nurses working at the University of Gondar and Tibebe Gion Teaching Specialized Hospitals, Amhara Region, Northwest Ethiopia.

This study was obtained findings as low prevalence of EBP, and associated factors eligible for EBP were educational level, attitude of Nurses towards EBP use, administrative support, and Preferred available information sources.

In this study, EBP among nurses of the study settings were found to be 47%. Only 207(41%), 174(34%), and 57 (11%) apply/use EBP in their clinical practice monthly, weekly, and always or daily respectively. This finding was consistent with the study done in Tikur Anbesa hospital in Addis Ababa, in which 57.6% of participants applied EBP. Of them, 64 (52.8%), 38 (31.4%), and 19 **(**15.7%**)** applied EBP sometime, usually, and always respectively (**10**). This was inconsistent with the finding of Nigeria (**22**) where 55.5% and 30.9% of nurses sometimes and often used EBP respectively. This suggests nurses working in the current study area were less likely to use EBP than other countries. It may be due to the Nurse's tendency to use more traditional resources such as colleagues and other medical professionals than up-to-date resources such as electronic resources and journals to make clinical decisions.
This result was also confirmed by the in-depth interview**,** *"Since there are no guidelines available for practicing evidence-based nursing in my workplace, I am not working my job based on the facts that are supported by updated best evidence, I only job by sharing the problems with my colleague's opinions " (G STA>10) (F1)*
"I only work with my knowledge that I have gained from my education, and sometimes I use Google to update my practice records". (G STA 5- 10) (M1)
"… No hospital library, updated guidelines, motivation, enough training, and no enough computers for nurses for updating themselves." (G STA 5–10) (F2)

The odds of EBP among nurses with MSc and above were 2.148 times higher (AOR= 2.15, 95%CI= (1.15-4.02)) when compared to BSc nurses (Table: 3 see Additional file 1). This is a contrast with the study of Nigeria (**22**) in which professional qualification has no relationship with the use of good EBP but analogous with the finding of a study

done in Ethiopia (**16**). It suggests that good EBP was most often used by nurses who had higher qualifications than by lower qualifications. This may be because the educational level MSc and above are more technically oriented, improving quest techniques, or being more open to the introduction of EBP into their curricula and teaching programs.

The attitude of nurses towards the integration of evidence-based practice in their clinical practice accounted for 72%. This finding was similar to the study conducted In South Africa 75% (**23**) and Singapore 64% (**19**). In our finding, the odds of EBP among nurses having a favorable attitude was 1.80 times higher (AOR=1.80, 95%CI= (1.24-2.62)) when compared to unfavorable attitudes. This finding agrees with the studies conducted in South Africa (**19**). This may be due to the difference in nurses' qualification level, work experience, and training which have the potential to significantly enhance beliefs and attitudes about EBP. Nurses with a higher level of qualification have better knowledge, which improves their attitude towards EBP.

This result was also confirmed by the in-depth interview in which most of the respondents suggest *"It is important for quality care, but the workload, lack of knowledge and training make us follow previous experience or rely on opinions of experts."*

The odds of EBP among nurses having administrative support were 1.89 times higher (AOR=1.89, 95%CI= (1.22-2.91)) when compared to having no support. This is in line with the study conducted in Ethiopia (**10**). Other studies also identified managers, not only as a key factor for the generation and implementation of EBCP but also for the creation of a good research environment (**24**).

It was confirmed by the qualitative as:

Most participants believed that “*they need training and budget support for evidence”*

“Training center has to be established to give training for us on how to integrate research evidence in our practices and to develop guidelines for practice, thus serving as a source for guiding our health interventions” (G STA<5) (F3)

“We clinical staff need our higher administrator's help in conducting clinical research because we know our problem better than academic staff, and if we get the chance to

perform research on our clinical issue, we can do and incorporate research evidence into our daily practices". (G STA 5–10) (M2)

The odds of EBP among nurses Preferred available information sources were 2.32 times higher (AOR: 2.32, 95%CI: 1.58-3.39) when compared to counterpart. This study is consistent with prior studies (**25, 26**) which found that human information sources and sources of print information were used more frequently by them. This was presumably because of their higher usability and the ease of using them and having less Internet access, lowers device use, and less participation in online activities. It was worth noting that the use of electronic information sources by nurses was the least common, given the fact that they have low ability, interpersonal and technical competence of information searching skill of the latest research information.

It was confirmed as:

The majority of nurses said**,** *"We need training on how to use evidence from journals and databases, and how to search for literature to provide effective interventions in health care".*

"…*their desire to use the information is as poor as possible because the profession does not do as much as the profession allows, the nurse professional does not have a culture of communicating and learning one another. So, we need training on how to use evidence from and how to search for literature from databases to provide effective health care interventions."* (G STA >10) (M3)

Strength

- This study used both quantitative and qualitative research design to analyze some factors associated with EBP
- A pretest was conducted to test the tools and overall procedures of the study
- Unlike other studies, it has a 95.7% response rate.
- This study was conducted in two of the Amhara regional state teaching specialized hospitals (the University of Gondar and Tibebe Gion), which enables the generalizability of the results to other teaching specialized hospitals of Ethiopia.

Limitations

- To compare in a local context, no more previously studied researches were available in Ethiopia.

IMPLICATIONS OF THE STUDY

The findings of this study have implications for practice, education, policy, and research. It will be supremely significant to the policymakers and more specifically to the nursing and health care professionals to provide quality of care in meeting the needs of patients and families as a whole specifically it improves patient outcomes.

CONCLUSIONS AND RECOMMENDATIONS

This study concluded that a small number of nurses applied EBP to a high level. The implementation of evidence-based practice was associated with educational level, attitude towards EBP, administrative support, and the availability of information resources and it was confirmed by the qualitative study. There has to be an intervention program to facilitate the implementation of evidence in nursing practice by the respective stakeholders (Ethiopian Federal Ministry of Health, Amhara Regional Health Bureau, educators of nursing education, and hospital administration of Amhara Region Teaching Referral public hospitals).

Training should be conducted for nurses on the implementation of evidence-based practice by Amhara Region Teaching Referral public hospitals in collaboration with NGOs, the Ethiopian Federal Ministry of Health, and the Amhara Regional Health Bureau. Resources necessary to implement evidence-based nursing practice should be provided by hospital administration in collaboration with the Ethiopian Federal Ministry of Health, Amhara Regional Health Bureau, and other NGOs. Intervention programs concerning organizational communication with issues on evidence-based practice implementation should be done together with nurses to create supportive staff by the nursing administration of the study setting. Since there is a gap of attitude towards evidence-based nursing practice curriculum planners should take into consideration to include the principle of evidence-based nursing practice in Ethiopian nursing education programs especially in the undergraduate nursing education curriculum.

Abbreviations

AOR: adjusted odds ratio, CI: Confidence interval, COR: Crude odds ratio,

EBP: Evidence-Based Practice, G ADM: Group of nurses with Administrative responsibilities, G TEAM: Group of nurses with team leader responsibilities,
G STA<5: Group of Staff nurses with less than 5 years of professional experience, G STA5–10: Group of Staff nurses with professional experience ranging from 5 to 10 years and G STA > 10: Group of Staff nurses with more than 10 years of professional experience.

Declarations:

Ethics approval and consent to participate

The study protocol was reviewed and approved by an institutional ethical review committee of the University of Gondar and written informed consent was obtained from each study participants. Permission letter also obtained from each Hospitals. All the data used in this manuscript were privately available and confidentiality was maintained anonymously.

Consent for publication

Not applicable

Competing interests

The authors declare that they have no competing interests.

Funding

No funding.

Availability of data and materials

The datasets generated and/or analyzed during the current study will be available upon reasonable request from the corresponding author.

Authors' contributions

AB selected the title, developed the proposal, collect the data, analyzed the data, interpreted the results, and prepared the manuscript. TM and MA assisted the design,

comment, and approve the proposal, thesis, and the manuscript. AI assisted in the preparation of the manuscript and commented and edited the manuscript. All authors have read and approved the manuscript.

Acknowledgments

The authors acknowledge the Institute of Public Health, College of Medicine and Health Science, University of Gondar. Our thanks also extended to the participating nurses for their cooperation in this study.

Authors' information

Abebe Birhanu Degu (BScN, MPH in Health Informatics); **Tesfahun Melese Yilma** (BSc, MPH, Ph.D.); **Miftah Abdella Beshir** (BSc, MPH); **Anushia Inthiran** (BSc, MSc, PhD).

Authors' Detail

[1,] Department of Health Informatics, College of Medicine and Health Sciences, University of Gondar, Gondar, Ethiopia

[2] Department of Accounting and Information Systems, College of Business and Law, University of Canterbury, Meremere, New Zealand

References

1. Melnyk BM, Fineout-Overholt E. **Evidence-based practice in nursing & healthcare: A guide to best practice**: *Lippincott Williams & Wilkins*; 2011. 208-224.
2. Banning MJJoCN. Conceptions of evidence, evidence-based medicine, evidence-based practice, and their use in nursing: independent nurse prescribers' views. 2015;14 (4):411-417.
3. Tranmer Ja, Squires S, Brazil K, Gerlach J, Johnson J, Muisiner D, et al. **Factors that influence evidence-based decision-making**. 1998; 5:3-92.
4. Kristensen N, Nymann C, Konradsen HJBhsr. I**mplementing research results in clinical practice-the experiences of healthcare professionals in Denmark**. *BMC Health Service Research*. 2015;16(1):48.
5. Mokhtar IA, Majid S, Foo S, Zhang X, Zheng Y-L, Chang Y-K, et al. **Evidence-based practice and related information literacy skills of nurses in Singapore: an exploratory case study**. *Malaysian Journal of Literacy and Information Science*. 2012; 18 (1):12-25.
6. Parse RR. **Research as evidence: Many meanings**. *Los Angeles*: *Sage Publications Sage CA: Los Angeles, CA*; 2013. p. 20-24.
7. Williams B, Perillo S, Brown T. **What are the factors of organizational culture in health care settings that act as barriers to the implementation of evidence-based practice? A scoping review**. *Nurse education today*. 2015; 35(2):e34-e41.
8. Labrague LJ, McEnroe-Pettite D, Taras K, D'Souza MS, Fronda DC, Mirafuentes EC, et al., editors. **Predictors of evidence-based practice knowledge, skills, and attitudes among nursing students**. Nursing forum; 2019: *Wiley Online Library*.
9. Naidoo JR, Adamu A. **Exploring the perceptions of registered nurses towards evidence-based practice in a selected general hospital in Nigeria**. *Africa Journal of Nursing and Midwifery*. 2015; 17(1):33-46.
10. Hadgu G, Almaz S, Tsehay SJAJNS. **Assessment of nurses' perceptions and barriers to evidence-based practice in Tikur Anbessa specialized hospital Addis Ababa Ethiopia**. *ResearchGate*. 2015; 4(3):73-83.
11. Mutisya AK, KagureKarani A, Kigondu C. **Research utilization among nurses at a teaching hospital in Kenya**. *Journal of caring sciences*. 2015; 4(2):95.

12. Davis DA, Taylor-Vaisey A. **Translating guidelines into practice: a systematic review of theoretic concepts, practical experience, and research evidence in the adoption of clinical practice guidelines**. *Cmaj*. 1997; 157(4):408-416.
13. Ross JJJoPN. **Information literacy for evidence-based practice in peri-anesthesia nurses: readiness for evidence-based practice in the USA.** *Journal of peri-anesthesia Nursing* 2010; 25(2):64-70.
14. Polit DF, Beck CT. **Nursing research: Generating and assessing evidence for nursing practice**: *Lippincott Williams & Wilkins*; 2008.
15. Morales-Asencio J, Sesé AA, Bennasar MV, Artigues GV, Perelló CC. **Nursing practice settings and competence to incorporate evidence into decisions: analysis of the situation in the Balearic Islands (Spain)**. *Gaceta sanitaria*. 2011; 25(3):191-197.
16. Dereje B, Hailu E, Beharu MJAPDS. **Evidence-based Practice Utilization and Associated Factors among Nurses Working in Public Hospitals of Jimma Zone Southwest Ethiopia: A Cross-Sectional Study**. *General Medicine*. 2019; 7:321.
17. Saunders H, Vehvilainen-Julkunen K. **Nurses' Evidence-Based Practice Beliefs and the Role of Evidence-Based Practice Mentors at University Hospitals in Finland**. Worldviews on evidence-based nursing. 2017; 14(1):35-45.
18. Beshir MA, Woreta SA, Kebede M. **Evidence-based practice among health professionals in hospitals of Northwest Ethiopia: a cross-sectional study**. International journal of evidence-based healthcare. 2017; 15(4):161-170.
19. Majid S, Foo S, Luyt B, Zhang X, Zheng Y-L, Chang Y-K, et al. **Adopting evidence-based practice in clinical decision making: nurses' perceptions, knowledge, and barriers**. *Singapore Journal of the Medical Library Association*. 2011; 99(3):229.
20. Shifa F, Evans D, Bradley HJAiN. **Nurses' perceptions of barriers and facilitators to implementing EBP in the Maldives**. *Hindawi Publishing Corporation*. 2014; 2014:14.
21. SHAPIRO SS, Wilk MB. **An analysis of variance test for normality (complete samples)†in Great Britain**. *Biometrika*. 1965; 52(3-4):591-611.

22. Naidoo JR, Adamu AJAJoN, Midwifery. **Exploring the perceptions of registered nurses towards evidence-based practice in a selected general hospital in Nigeria**. 2015; 17(1):33-46.
23. Solomons NM, Spross JAJJonm. **Evidence-based practice barriers and facilitators from a continuous quality improvement perspective: an integrative review**. 2011; 19(1):109-120.
24. Strout TD, Lancaster K, Schultz AA. **Development and implementation of an inductive model for evidence-based practice: A grassroots approach for building evidence-based practice capacity in staff nurses**. *Nursing Clinics*. 2009; 44(1):93-102.
25. Jones J, Schilling K, Pesut D. **Barriers and benefits associated with nurses information seeking related to patient education needs on clinical nursing units**. *The open nursing journal*. 2011; 5:24.
26. Jordan P, Bowers C, Morton DJSAJoCC. **Barriers to implementing evidence-based practice in a private intensive care unit in the Eastern Cape**. 2016; 32(2):50-54.

Click here to access/download;Figure;Additional file 2. Figure files.docx

Figure Files:

List of figures presented in the study of EBP and its associated factors among nurses working atteaching specialized hospitals in Northwest Ethiopia, 2020

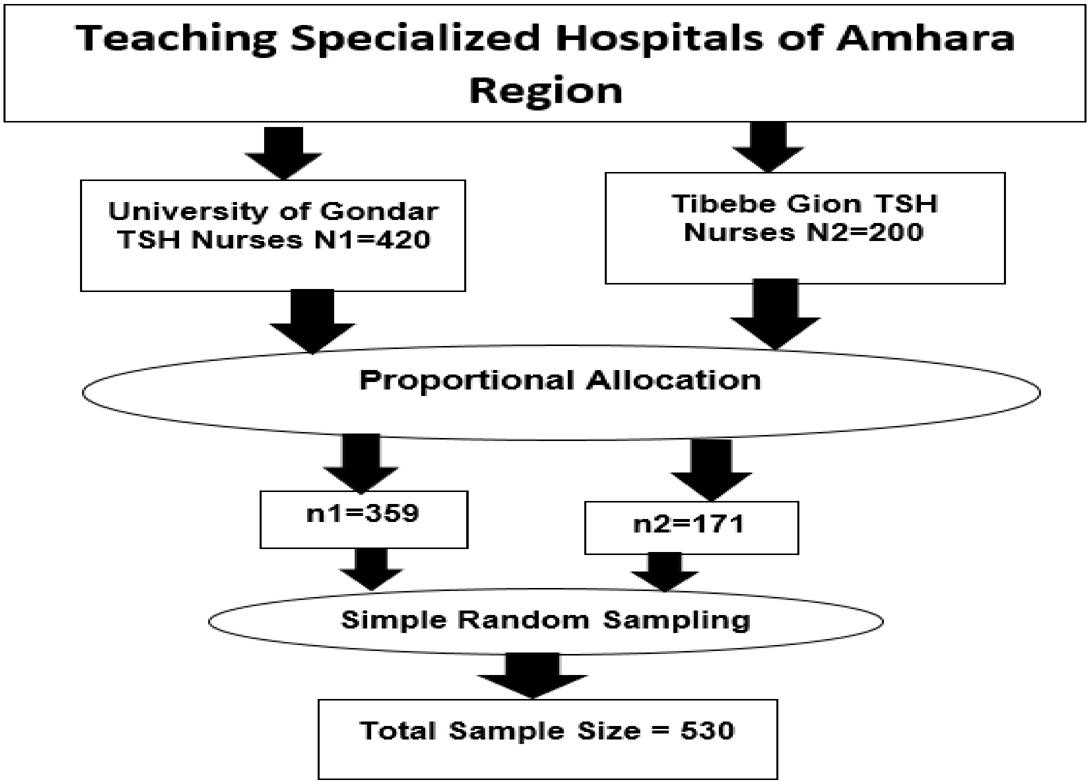

Fig. 1: *Sampling procedure of the study of Evidence-Based Practice among nurses at the University of Gondar and Tibebe Gion Teaching Specialized Hospitals, 2020*

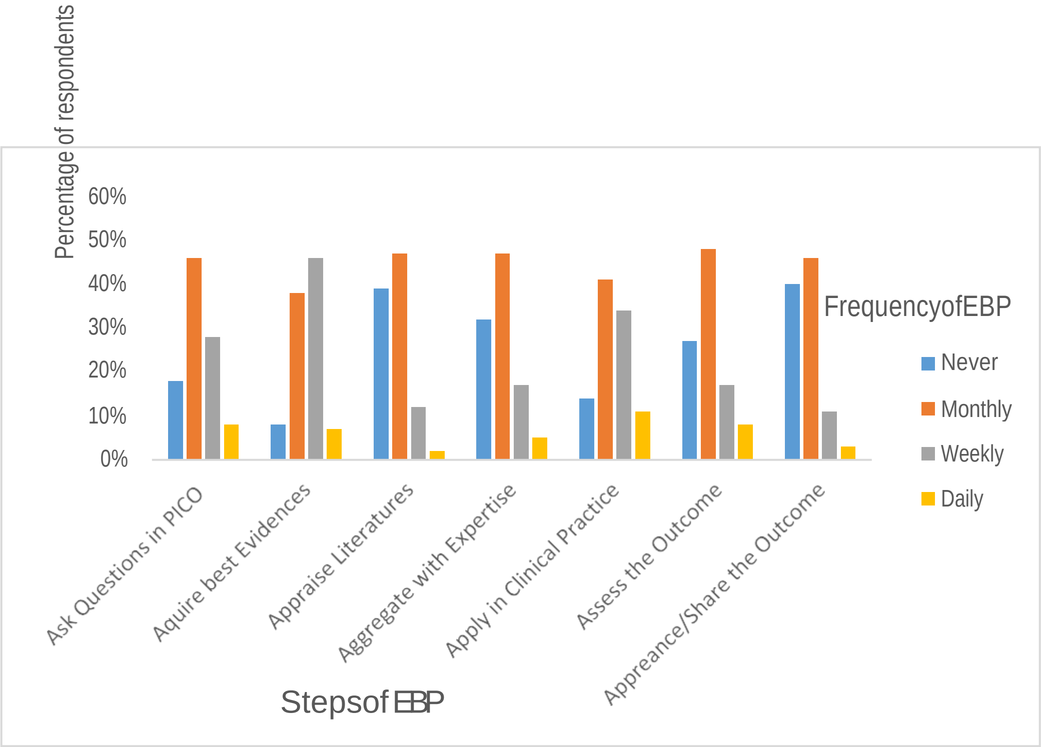

Fig. 2: *Evidence-Based Practice among nurses at the University of Gondar and Tibebe Gion Teaching Specialized Hospitals by nurses in 2020.*

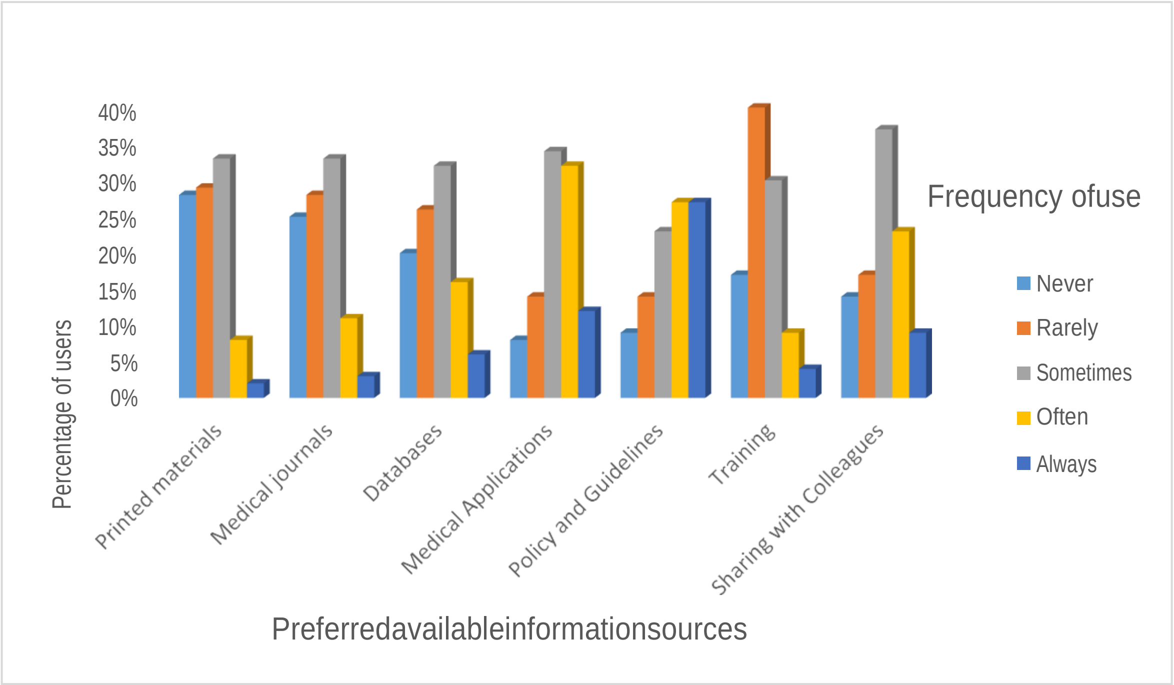

Fig. 3: *Preferences on Sources of Evidence-Based Practice among Nurses in University of Gondar and Tibebe Gion Teaching Specialized Hospitals, 2020 (n=507)*

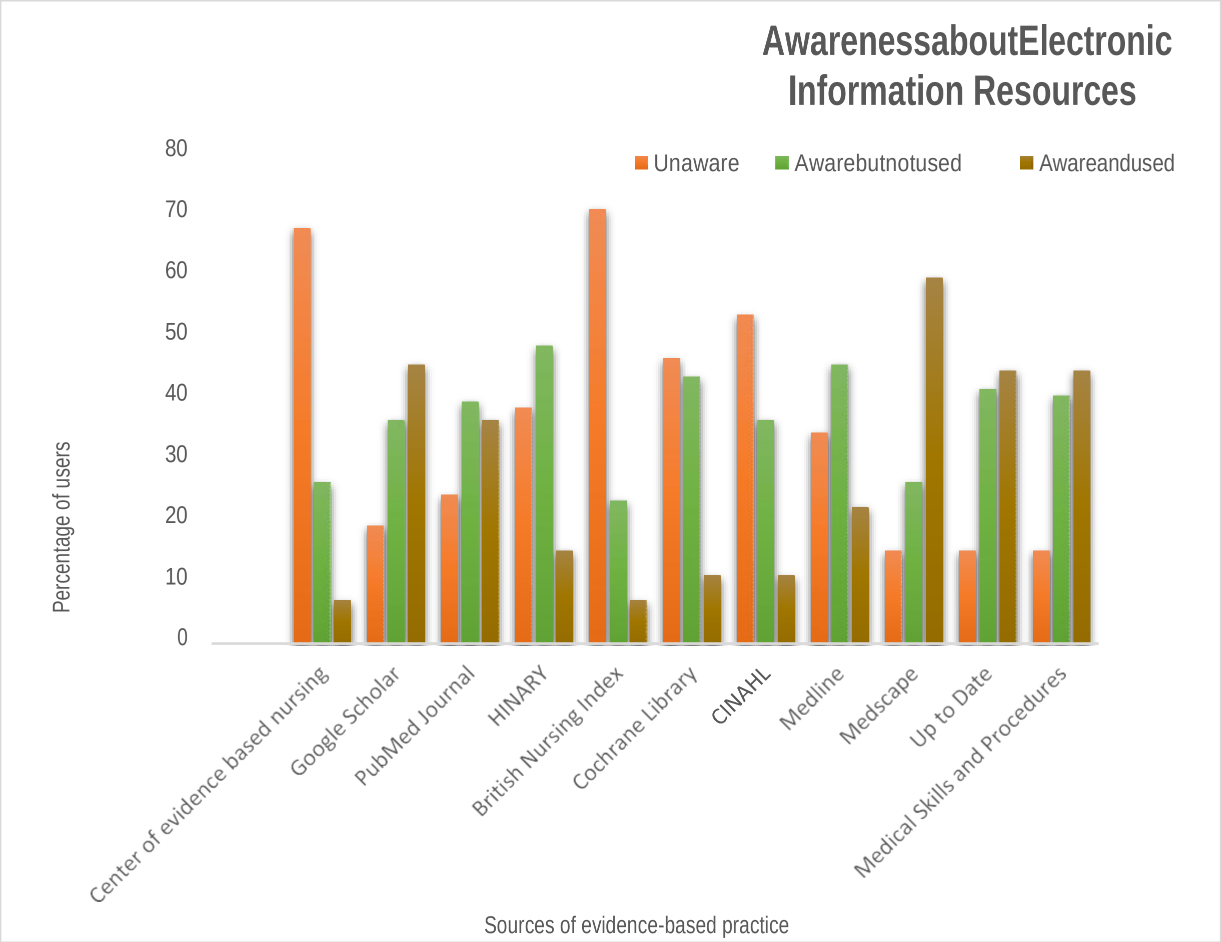

Fig. 4: *Awareness about Electronic Information Sources of Evidence-Based Practice among Nurses in University of Gondar and Tibebe Gion Teaching Specialized Hospitals, 2020 (n=507)*

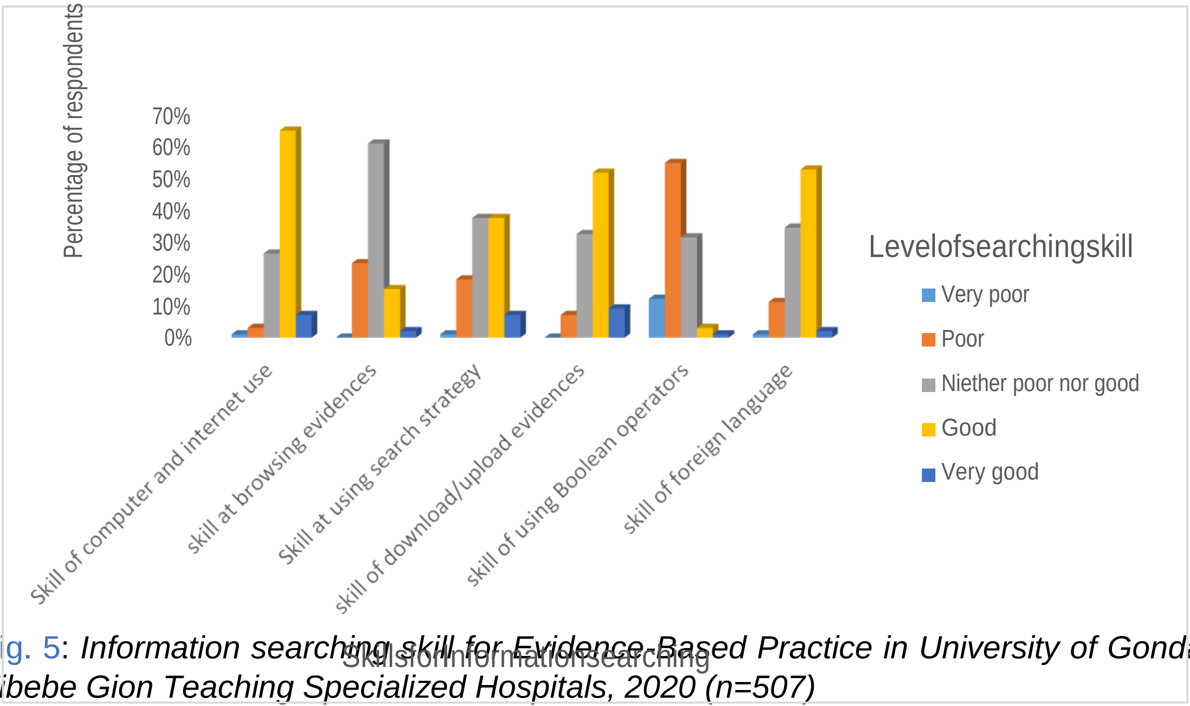

Fig. 5: *Information searching skill for Evidence-Based Practice in University of Gondar and Tibebe Gion Teaching Specialized Hospitals, 2020 (n=507)*

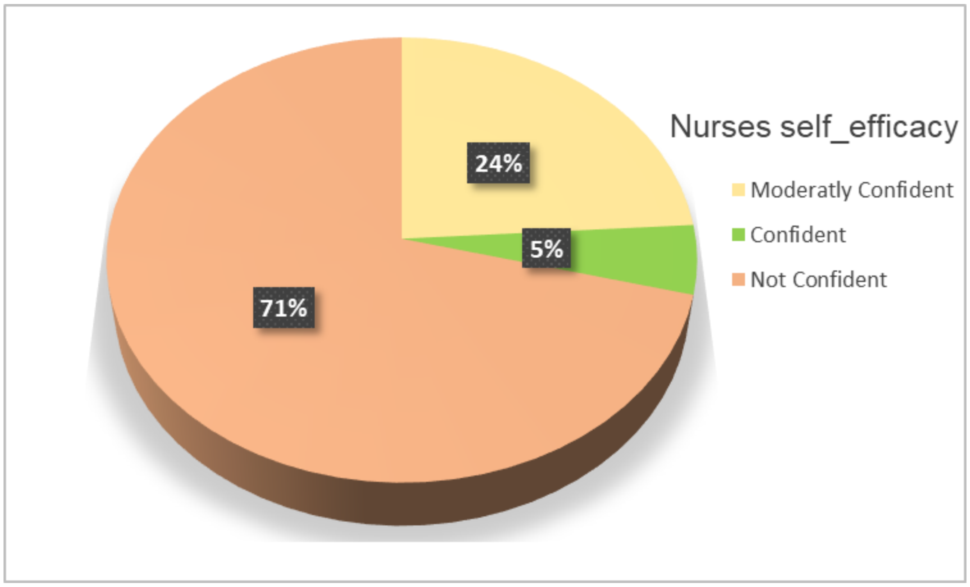

Fig. 6: *Nurses Self-Efficacy in Evidence-Based Practice at the University of Gondar and Tibebe Gion Teaching Specialized Hospitals, 2020 (n=507)*

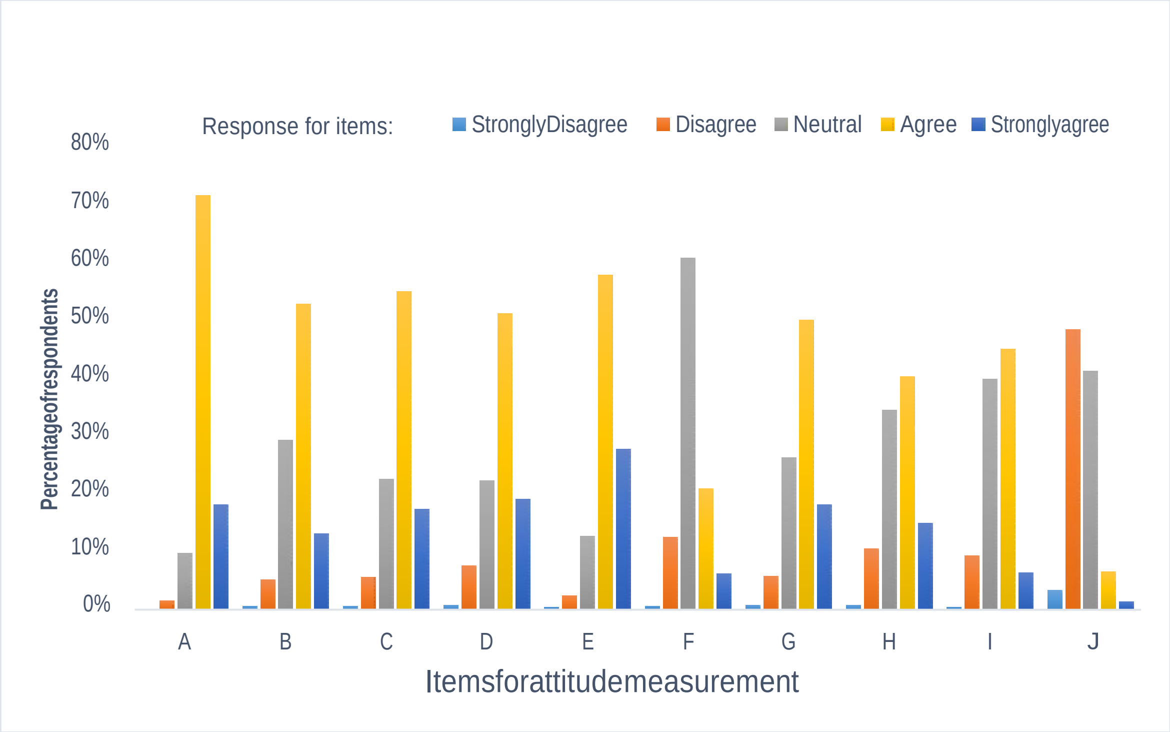

Fig. 7: *Self-reported attitudes towards evidence-based practice among Nurses in the University of Gondar and Tibebe Gion Teaching Specialized Hospitals, Ethiopia 2020.*

Keys: A= Research and literature findings are useful in day-to-day practice B= application of EBP is necessary, C= I need to increase the use of evidence, D= I am interested in learning and improving EBP skills, E= EBP improves the quality of patient care, F= my reimbursement rate will increase if I use EBP, G= EBP helps in decision making, H= my workload is not too high to keep up-to-date, I= Critical appraisal is a necessary step and J= I have access to the best resources needed for EBP

Click here to access/download
Supporting Information
Additional file 1. Table files.xml

Click here to access/download
Supporting Information
Additional file 3. SPSS dataset.csv
